# Substance P Saliva Reduction Predicts Pharyngeal Dysphagia in Parkinson's Disease

**DOI:** 10.3389/fneur.2019.00386

**Published:** 2019-04-16

**Authors:** Jens Burchard Schröder, Thomas Marian, Inga Claus, Paul Muhle, Matthias Pawlowski, Heinz Wiendl, Sonja Suntrup-Krueger, Sven G. Meuth, Rainer Dziewas, Tobias Ruck, Tobias Warnecke

**Affiliations:** Department of Neurology, University Hospital Münster, Münster, Germany

**Keywords:** Parkinson's disease, substance P, dysphagia, biomarker, neurodegeneration

## Abstract

**Introduction:** Although patients with Parkinson's disease (PD) often suffer from oropharyngeal dysphagia, knowledge about the underlying pathophysiological mechanisms is limited. Substance P (SP) is a localization-independent neurotransmitter of the entire nervous system. Reduced levels of SP were found in saliva of patients with impaired cough reflex and in advanced stages of PD. The aim of the study was to investigate SP in PD patients in order to gain further insights into the underlying pathophysiology of PD-related dysphagia and to evaluate the potential of SP as a biomarker for early dysphagia.

**Methods:** Flexible endoscopic evaluation of swallowing (FEES) was used to objectively assess pharyngeal swallowing function. From a cohort of 105 consecutive PD patients 20 subjects were recruited: in 10 of them pharyngeal dysphagia was excluded by FEES, the other 10 subjects showed signs of early pharyngeal dysphagia defined as hypopharyngeal sensory deficit with mild to moderate vallecular residues after swallowing solid consistencies. Analysis of the Substance P level in saliva of the 20 included PD patients was performed in the clinical on state condition by ELISA-type immunoassay. Significant differences were calculated by using the Mann-Whitney test.

**Results:** Twenty PD patients with a mean age of 69.5 ± 12.5 years (8 female) were included in the study. No significant differences were found regarding gender, age, UPDRS III, Hoehn and Yahr stage, disease duration, and Levodopa equivalent dose between the non-dysphagic and dysphagic subjects. Dysphagia was mainly characterized by unrecognized residues in the valleculae without any aspiration risk for all of the tested consistencies in FEES and was thereby scored as mild in all cases. Saliva SP concentrations were significantly lower in PD patients with pharyngeal dysphagia compared to those with a normal pharyngeal swallowing function (9,644 vs. 17,591 pg/mL; *p* = 0.001).

**Conclusion:** Reduced saliva SP concentrations may predict early pharyngeal swallowing dysfunction in PD patients. This finding supports the hypothesis that an impaired SP mediated neurotransmission has a significant impact for the development of dysphagia in PD patients. Larger studies are needed to confirm SP as a clinical useful biomarker for early detection of PD-related dysphagia.

## Introduction

Following Alzheimer's disease, Parkinson's disease (PD) is the second most common neurodegenerative disorder (1, 2). Oropharyngeal dysphagia is a clinically relevant symptom in affected patients as the majority of PD patients will suffer from neurogenic dysphagia during the course of their disease (3–6). In addition to consecutive malnutrition, dehydration and insufficient medication intake, neurogenic dysphagia leads to loss of quality of life for affected patients and aspiration pneumonia, which is the leading cause of death in Parkinson's patients (4, 7–9).

However, clinical diagnosis of dysphagia in PD remains challenging. Particularly in early disease stages affected patients are usually unaware of their swallowing dysfunction and therefore do not report spontaneously about swallowing problems (10, 11). Furthermore, pharyngeal swallowing function cannot be assessed sufficiently by clinical neurological examination resulting in a significant delay of uncovering dysphagia in these patients (11–13). When more elaborate instrumental tools for swallowing evaluation like flexible endoscopic evaluation of swallowing (FEES) or videofluoroscopic swallowing study (VFSS) are systematically applied, dysphagia was found to be present in more than 50% of subjectively asymptomatic PD patients (14). However, access to FEES and VFSS is limited in many institutions.

Furthermore, the underlying pathophysiology of dysphagia in PD and particularly the role of substance P (SP) is still barely understood (15). SP is an ubiquitary neuropeptide in the nervous system (16). It mediates the response to local stimuli in the pharyngeal mucosa and thereby enhances the swallow and cough reflex (17–19). In a cohort of elderly patients with aspiration pneumonia, sputum levels of SP were found to be reduced (20). Probably due to a reduced concentration of SP in the saliva of patients in advanced stages of PD, protective reflexes with consecutive silent aspiration were observed (21, 22). However, it is so far unknown if substance P reduction is already present in PD patients with early pharyngeal dysphagia not showing any risk of aspiration.

The purpose of the present study was to investigate Substance P as a potential biomarker for early detection of pharyngeal dysphagia in PD and thereby gain further insights into pathophysiology.

## Material and Methods

### Protocol Approval, Registration, and Patient Consent

All patients were prospectively recruited from the movement disorder unit at the Department of Neurology, University Hospital Münster, Germany. Written informed consent was obtained from each subject after the nature of the study was explained in accordance to the principles of the declaration of Helsinki. The local ethics committee of the medical faculty at the University of Muenster approved the protocol of the study (2014-624-f-S).

### Participants

From a cohort of 105 consecutive PD patients, who did not have subjective swallowing impairment and were evaluated for dysphagia with FEES, as part of the baseline dysphagia assessment in our clinic, 20 subjects were recruited between January 2017 and March 2018. Participants had to be on stable medication regimen and all examinations were done in the clinical “ON” phase. Exclusion criteria for this study were concomitant diseases that may cause neurogenic dysphagia, autoimmune diseases, anti-inflammatory co-medication (e.g., cytotoxic agents, steroids, non-steroid analgesia), sedatives, or evidence of an acute systemic inflammatory process at the time of Substance P measurement (elevated erythrocyte sedimentation rate above 25 mm/h, C-reactive protein above 0.5 mg/dL, or leukocytes above 11 × 103/μL). Patient characteristics are described in detail in Table 1.

**Table 1:** Subject characteristics and clinical features.

	**All**	**Dysphagia +**	**Dysphagia –**	***p*-value**
Number of patients (M/F)	20 (12/8)	10 (8/2)	10 (4/6)	0.068
Mean age (year)	69.5 ± 12.5	71.7 ± 11.8	67.2 ± 13.3	0.172
Disease duration (year)	8.35 ± 5.8	10.9 ± 6.5	5.8 ± 3.7	0.403
Hoehn and Yahr stage^a^	2.4 ± 0.8	2.8 ± 0.6	2.0 ± 0.7	0.698
UPDRS III, points^a^	16.5 ± 4.7	18.2 ± 4.8	14.8 ± 4.1	0.120
L-Dopa-Dose-Equivalent, mg	768.8 ± 276.4	849.2 ± 264.7	688.3 ± 277.1	0.199
Saliva quantity, microliters	792.9 ± 122.5	752.1 ± 108.1	833.8 ± 127.5	0.287
PPI intake (*n*)	7	4	3	0.180

*UPDRS, Unified Parkinson Disease Rating Scale*.

a *At clinical “On”-stage*.

### Dysphagia Assessment

In all participants, Dysphagia was examined with fiberoptic endoscopic evaluation of swallowing in the on-state condition. FEES was performed in accordance with the standard protocol proposed by Langmore et al. as described in detail elsewhere (23).

Equipment consisted of a 3.1 mm-diameter flexible fiberoptic rhinolaryngoscope (11101RP2, Karl Storz, Tuttlingen, Germany), a light source and camera (rpCam-X, rpSzene®, Rehder/Partner, Hamburg, Germany), a color monitor (WMP-226, Wincomm, Taiwan) and a video recorder (AUCC2WV3F, Computar, CBC Group, Japan).

All PD patients were given nine test boluses in a standardized order. First, they received 8 ml of pudding (green jelly), second 5 ml blue-dyed liquid and finally three trials of white bread with the size of approximately 3 × 3 × 0.5 cm. During all swallowing tasks, the following parameters of swallowing function were assessed: premature spillage, material that enters the hypopharynx unintentionally from the oral cavity before the pharyngeal swallow was initiated, penetration-aspiration events or residues. When residues in FEES were observed, patients were asked explicitly if they perceived any foreign body sensation in their throat.

Swallowing dysfunction was classified with a FEES-based 4-grade dysphagia severity scale that has previously been developed and published: 0 = no relevant dysphagia, 1 = mild dysphagia (premature spillage and/or residues, but no penetration/aspiration events), 2 = moderate dysphagia (penetration/aspiration events with one consistency), 3 = severe dysphagia (penetration/aspiration events with two or more consistencies) (24–26). Substance P was collected from patients with either no signs of pharyngeal dysphagia (group 1), or endoscopic signs of early pharyngeal dysphagia (group 2). Dysphagia was defined as mild to moderate vallecular residues being present after swallowing solid consistencies (27). Patients showing an increased aspiration risk for any of the tested consistencies were excluded.

### Sample Collection and Analysis:

Between 09 and 11 a.m., about 2 h after the intake of their regular dopaminergic medication, sputum was collected. Two salivettes were placed into the patient's oral cavity for 1–3 min. Directly after collecting the saliva, the salivettes were centrifuged at 4,110 g for 5 min. Supernatants were stored in a deep freezer at −20°C until further analysis. Substance P levels were assessed by a commercially available competitive ELISA-type immunoassay according to the manufacturer's instructions (SP Immnunoassay, catalog no. KGE007; R&D Systems, Minneapolis, MN, USA).

### Statistics

Statistical analysis was performed using SPSS Version 25.0. All data are reported as mean ± standard deviation, and the pre-chosen significance level for all confirmatory tests was *p* < 0.05. Substance P-Level was analyzed by using the Mann-Whitney test, presuming a non-Gaussian distribution.

## Results

FEES was successfully performed in all 20 subjects (12 male, 8 female, mean age 68.6 ± 12.5 years), presenting with either no (*n* = 10) or early pharyngeal dysphagia (*n* = 10). No adverse events occurred during the FEES examinations. Table 1 summarizes patient's characteristics and clinical features of included patients. Gender and age were equally distributed in both study groups. In addition, no statistically significant differences were found regarding disease duration, L-Dopa equivalent doses, UPDRS III, and Hoehn & Yahr stages. PPI-intake was equally distributed between both groups.

Unnoticed pharyngeal residues were present in 10 patients (Group 2). They were located in the vallecular space in all subjects and, to a lesser extent in the pyriform sinuses (30%). Premature spillage and penetration/aspiration events were not observed for any consistency, i.e., liquid, semisolids, solids.

**Figure 1:**
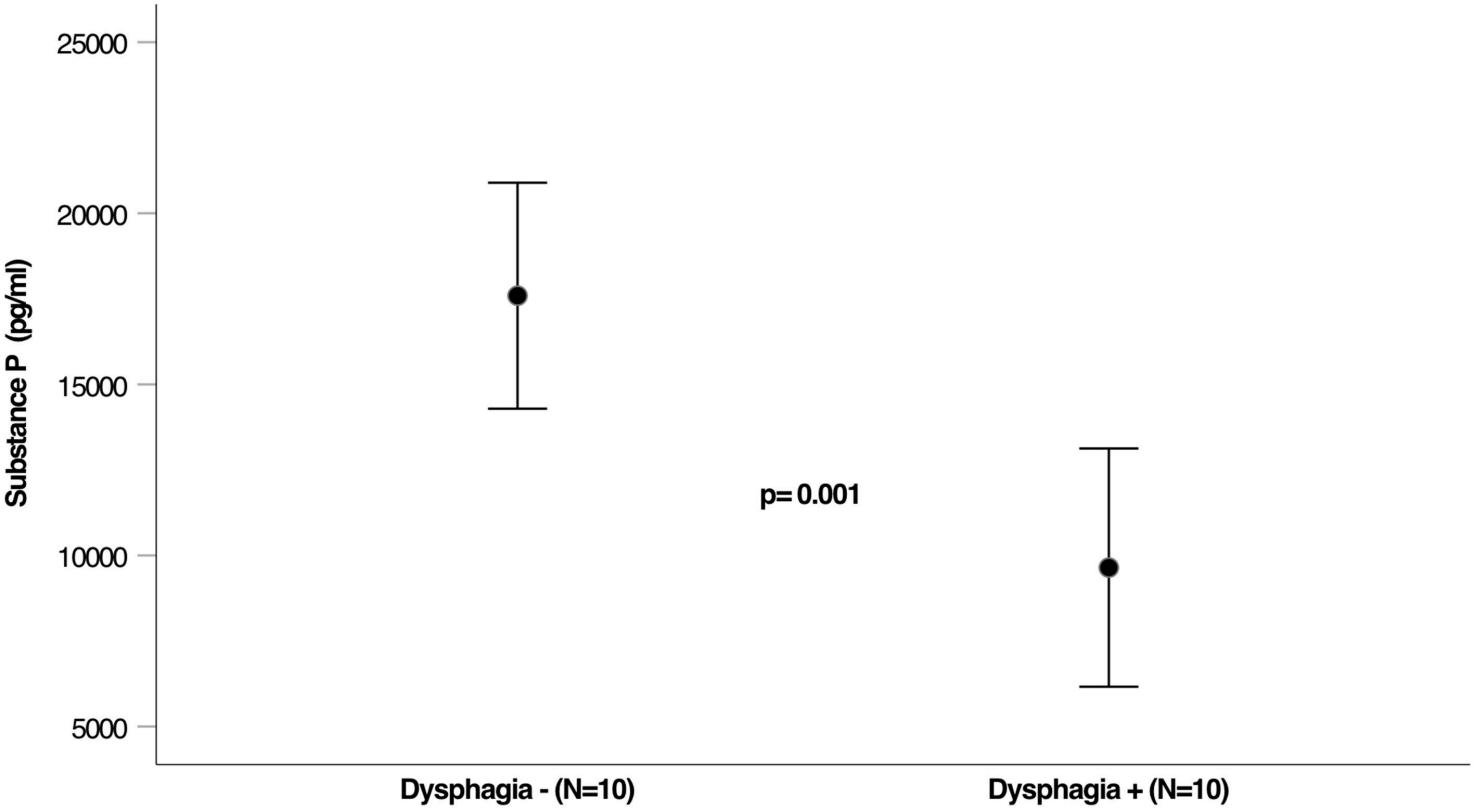
Levels of Substance P in saliva of dysphagic and not dysphagic PD patients.

Figure 1 shows the concentration of SP in sputum among the 2 groups. Sputum SP concentrations were significantly lower in patients with dysphagia compared to those in control subjects (9,644 vs. 17,591 pg/mL; *p* = 0.001; see Figure 1).

## Discussion

This prospective study analyzed the saliva concentrations of SP in PD patients with early pharyngeal dysphagia compared to PD patients without any pharyngeal swallowing impairment. The study's main finding is that early pharyngeal dysphagia in PD patients is associated with reduced levels of Substance P in patient's saliva.

PD-related dysphagia affects the oral, pharyngeal and the esophageal phase of swallowing and occurs in all stages of the disease (11, 28). Former studies have demonstrated a selective loss of vagal SP neurons in PD, potentially contributing to the emergence of esophageal motility disorders (29). In our study, also in patients with early pharyngeal dysphagia, as indicated by unnoticed significant pharyngeal residues, was associated with reduced SP concentrations. This finding broadens the results of a previous study, where significantly reduced levels of Substance P were found in PD patients with an impaired cough reflex sensitivity in a much more advanced stage of PD-related dysphagia. In early disease stages with by trend slightly lower Substance P levels cough sensitivity was not found to be impaired in this study (21). SP-immunoreactive fibers have been detected in the epithelium and basal membrane of pharyngeal mucosa, especially on the surface of the epiglottis (30). In this context, our findings could be another indication, that in early stages, loss of SP containing neurons in the pharyngeal mucosa may lead to pharyngeal hyposensitivity and merely incipient pharyngeal dysphagia.

Current diagnostic workup of suspected PD related dysphagia usually consists of questionnaires, swallowing assessment by speech language therapists and, in unclear cases, by using instrumental tools such as FEES or videofluoroscopic swallowing study (VFSS). Still, reliable screening methods are lacking (3, 27). FEES and VFSS are both considered to be the gold standard, but availability of instrumental assessment, especially in the outpatient setting, is often limited. In this context, the measurement of SP in saliva may have the potential to serve as a biomarker for the presence of clinically unrecognized pharyngeal dysphagia in early disease stages and thereby lead to earlier comprehensive dysphagia diagnosis and treatment.

Our study has several limitations that need to be addressed. First of all, PD patients were only assessed in one specific stage of their disease. Therefore, the results cannot be generalized over the entire PD population. For a possible use of substance P as a biomarker, in particular the establishment of reference values is necessary, which do not exist today. Furthermore, no follow-up of included patients was performed and, keeping in mind the basic experimental approach in the here presented pilot study, the sample size was rather low and no healthy controls were investigated. Any concomitant medication not excluded might have interfered with SP concentration. Impairment of motor function might as well have contributed to the emergence of dysphagia in our cohort. Furthermore, PD-related dysphagia is a complex symptom probably resulting from several central and peripheral mechanisms and thereby not being linked to one neurotransmitter system alone, and the exact localization of neurodegeneration, that leads to reduced SP release, cannot be identified in the clinical setting here (31).

In conclusion, this study showed for the first time, that reduced levels of SP occur in PD patients with signs of early pharyngeal dysphagia. Future studies should confirm this finding in larger cohorts. Moreover, agents like capsaicin, known to stimulate SP-release, should be investigated to assess their therapeutic potential by targeting the afferent sensory system within the swallowing network (32).

## Ethics Statement

Informed consent was obtained from each subject after the nature of the study was explained in accordance to the principles of the declaration of Helsinki. The local ethics committee of the medical faculty at the University of Muenster approved the protocol of the study (2014-624-f-S).

## Author Contributions

JS conceived, organized, executed the research project, and wrote the manuscript. TM reviewed the manuscript. PM reviewed the manuscript. IC reviewed the manuscript. MP reviewed the manuscript. HW helped to conceive the study and reviewed the manuscript. SS-K reviewed the manuscript. SM helped to conceive the study and reviewed the manuscript. RD reviewed the manuscript. TR conceived the research project, performed data analysis, and reviewed the manuscript. TW conceived and supervised the research project and reviewed the manuscript.

### Conflict of Interest Statement

The authors declare that the research was conducted in the absence of any commercial or financial relationships that could be construed as a potential conflict of interest.
